# The Interactions Between Autoinflammation and Type 2 Immunity: From Mechanistic Studies to Epidemiologic Associations

**DOI:** 10.3389/fimmu.2022.818039

**Published:** 2022-02-24

**Authors:** McKella Sylvester, Aran Son, Daniella M. Schwartz

**Affiliations:** Laboratory of Allergic Diseases, National Institute of Allergy and Infectious Diseases (NIAID), National Institutes of Health (NIH), Bethesda, MD, United States

**Keywords:** autoinflammation, autoinflammatory diseases (AID), allergy, type 2 immune response, type 2 immunity

## Abstract

Autoinflammatory diseases are a group of clinical syndromes characterized by constitutive overactivation of innate immune pathways. This results in increased production of or responses to monocyte- and neutrophil-derived cytokines such as interleukin-1β (IL-1β), Tumor Necrosis Factor-α (TNF-α), and Type 1 interferon (IFN). By contrast, clinical allergy is caused by dysregulated type 2 immunity, which is characterized by expansion of T helper 2 (Th2) cells and eosinophils, as well as overproduction of the associated cytokines IL-4, IL-5, IL-9, and IL-13. Traditionally, type 2 immune cells and autoinflammatory effectors were thought to counter-regulate each other. However, an expanding body of evidence suggests that, in some contexts, autoinflammatory pathways and cytokines may potentiate type 2 immune responses. Conversely, type 2 immune cells and cytokines can regulate autoinflammatory responses in complex and context-dependent manners. Here, we introduce the concepts of autoinflammation and type 2 immunity. We proceed to review the mechanisms by which autoinflammatory and type 2 immune responses can modulate each other. Finally, we discuss the epidemiology of type 2 immunity and clinical allergy in several monogenic and complex autoinflammatory diseases. In the future, these interactions between type 2 immunity and autoinflammation may help to expand the spectrum of autoinflammation and to guide the management of patients with various autoinflammatory and allergic diseases.

## Introduction

Diseases of immune dysregulation affect up to 40% of the global population and can have devastating consequences including organ failure and death (1, 2). Conceptually, disorders of immune activation are divided into three major categories. Autoimmune diseases are caused by inappropriate antigen-specific immune responses to self-antigens, and inflammation is largely promoted by lymphocytes (3). Allergic diseases are also mediated by inappropriate activation of lymphocytes, but the immune responses are against foreign antigens, or allergens (4, 5). By contrast, autoinflammatory diseases are caused by activated myeloid cells that mediate antigen-independent innate immune pathology (3). Although this is a useful conceptual framework, many autoimmune diseases are driven by a combination of innate and adaptive immune dysregulation (6, 7). The role of autoinflammatory pathways in autoimmune diseases has become a major area of investigation, uncovering novel interactions between innate and adaptive immunity (6, 7).

While the boundaries between autoimmunity and autoinflammation have become less clear over time, less work has been done on the intersection of allergy and autoinflammation. In general, autoimmune and autoinflammatory responses have been thought to primarily repress allergic inflammation, and vice versa (8). This is largely due to the Th1-Th2 (T helper 1 – T helper 2) paradigm, where Th1 and Th2 cells have counterregulatory roles. Th1 cells are associated with Type 1 immune responses, which are also characterized by activated myeloid lineage cells, and which are associated with autoimmunity and autoinflammation (9). However, over the past several decades it has become clear that autoinflammatory-associated cytokines and pathways can promote allergy-associated type 2 immune responses (5, 10). In this review, we explore the interactions between autoinflammation and type 2, or allergy-associated, inflammation. We begin by providing a brief overview of autoinflammation and type 2 inflammation, including the human diseases associated with both immune responses. We then review the role of autoinflammation-associated cytokines and pathways in type 2 responses, and the role of type 2 immune factors in autoinflammation. Finally, we summarize results from studies exploring the prevalence of type 2 clinical and immunologic phenotypes in patients with monogenic and complex autoinflammatory diseases.

## Part 1: An Overview of Autoinflammation and Type 2 Inflammation

### Autoinflammation Results From Inappropriate Innate Immune Activation

The concept of autoinflammatory disease was coined in 1999 to describe a group of immune dysregulatory diseases characterized by recurrent episodes of fever and systemic inflammation. In contrast to autoimmune diseases, autoinflammatory disorders are typified by constitutive activation of myeloid cells rather than antigen-specific T cell or B cell responses (3). Given the central role of myeloid cells in the innate arm of immune responses, the concept of “autoinflammation” was subsequently broadened to characterize primary disorders of the innate immune system. This approach was further advanced by the discovery of monogenic autoinflammatory diseases caused by mutations in genes critical for innate immune function (11–16).

**Figure 1:**
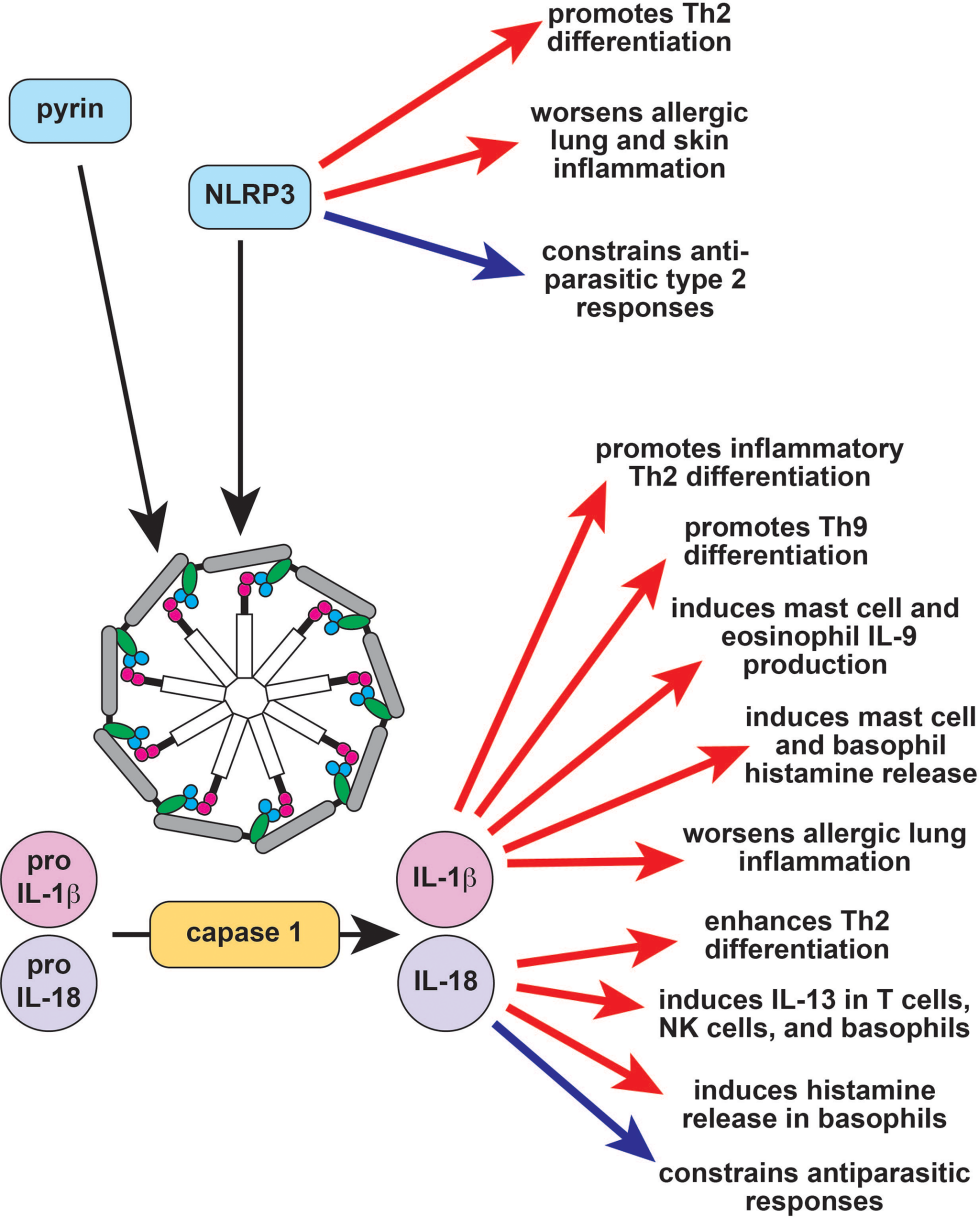
The role of the inflammasome in type 2 immune responses. Inflammasomes are large multimeric signaling molecules that process inactive pro-IL-1β and pro-IL-18 into their active forms. Constitutive activation of the pyrin inflammasome results in Familial Mediterranean Fever (FMF), while activation of NLRP3 causes the autoinflammatory disease cryopyrin-associated periodic fever syndrome (CAPS). NLRP3 induces Th2 differentiation through inflammasome-dependent and independent mechanisms (red arrows) but also acts as a brake on type 2 responses to parasites (blue arrow). IL-1b enhances allergic responses through a variety of effector cells (red arrows), while the effect of IL-18 is context-dependent (red and blue arrows). Th2, T helper 2; IL-9, interleukin 9; IL-13, interleukin 13; NK, natural killer.

One useful framework for characterizing monogenic autoinflammatory diseases is by the innate immunologic pathways that are dysregulated by disease-causing mutations. Many autoinflammation-associated genes are critical to the inflammasome and IL-1β production pathway (**Figure 1**). This includes the *MEFV* gene, which causes the prototypical autoinflammatory disease Familial Mediterranean Fever (FMF). Other examples of inflammasome-regulating genes and associated autoinflammatory diseases include *MVK* (hyper-IgD syndrome; HIDS), *NLRP3* (Cryopyrin-associated periodic fever syndrome; CAPS), *PSTPIP1* (Pyogenic arthritis with pyoderma gangrenosum and acne; PAPA), *WDR1* (periodic fever, immunodeficiency, and thrombocytopenia; PFIT), *IL1RA* (Deficiency of IL-1RA; DIRA), and *NLRC4* (Macrophage activation syndrome; MAS). Inflammasomes are innate immune sensors; upon activation, they form multimeric complexes that cleave the protease caspase-1, which in turn cleaves and activates IL-1β and IL-18. Consequently, inflammasomopathies are characterized by overproduction of IL-1β, and affected patients respond clinically to inhibitors of IL-1β and its receptor (3).

**Figure 2:**
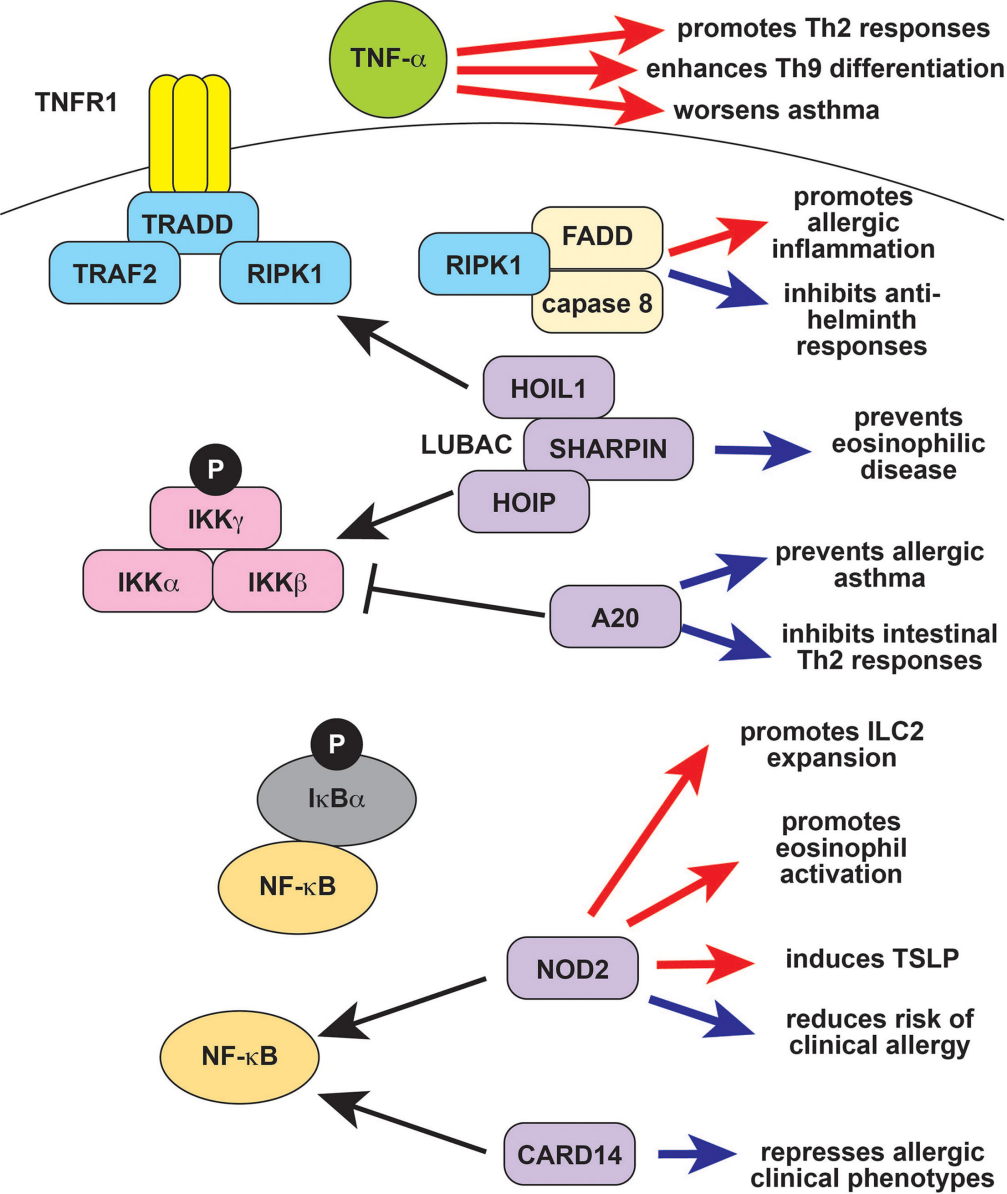
TNF-α and NF-κB signaling in type 2 immune responses. TNF-α exacerbates type 2 diseases like asthma in part by promoting Th9 and Th2 differentiation and function (red arrows). The protease caspase-8 forms the ripoptosome complex together with the TNF signaling molecule RIPK1 and FADD. The ripoptosome is regulates cell death, with caspase-8 and RIPK1 promoting apoptosis over necroptosis, so that defects in RIPK1 result in increased necroptosis and autoinflammation. The ripoptosome promotes type 2 responses in response to environmental allergens (red arrow) but can suppress type 2 responses to parasites (blue arrows). Ubiquitin editing proteins like A20 (*TNFAIP3*) and LUBAC (composed of HOIL-1, HOIP, and SHARPIN) modulate NF-κB signaling by targeting upstream molecules for activation and/or degradation. A20 negatively regulates NF-κB and also prevents allergic asthma as well as other type 2 responses (blue arrows). SHARPIN activates NF-κB, and deficiency results in eosinophilic tissue infiltration (blue arrow). The NF-κB signaling molecules CARD14 and CARD15/NOD2 also modulate type 2 responses. CARD14 prevents allergic disease, and deficiency results in clinical atopy (blue arrow). CARD15/NOD2 is reported to have both positive (red arrows) and negative (blue arrows) effects on type 2 immunity, and its role may be context-dependent. TNF-α, tumor necrosis factor alpha; RIPK1, Receptor Interacting Serine Threonine Kinase 1; FADD, Fas Associated *via* Death Domain; LUBAC, linear ubiquitin chain assembly complex; HOIL-1, Haem-Oxidized IRP2 Ubiquitin Ligase 1; HOIP, HOIL-1L Interacting Protein; SHARPIN, SHANK-associated RH-interacting protein; TNFAIP3, TNF-α induced protein 3; CARD14, caspase recruitment domain-containing protein 14; CARD15, caspase recruitment domain-containing protein 15; NOD2, nucleotide binding oligomerization domain-containing protein 2; Th2, T helper 2; IL-9, interleukin 9; IL-13, interleukin 13; IL-4, interleukin 4; NK, natural killer.

Another group of diseases is caused by mutations in the tumor necrosis factor (TNF)/NF-κB signaling pathway, which modulates innate and adaptive immune responses (**Figure 2**) (17). The prototypical example of TNF-receptor associated periodic fever syndrome (TRAPS) is caused by mutations in *TNFRSF1A*, although the pathogenesis of TRAPS is complex and includes TNF-independent mechanisms (18). Downstream of the TNF receptor, ubiquitin-editing enzymes like OTULIN and A20 negatively regulate NF-κB signaling; inactivating mutations cause the autoinflammatory diseases Otulipenia and HA20, respectively (19, 20). Gain-of-function mutations in the *NOD2* and *CARD14* genes also cause autoinflammation due to constitutive activation of NF-κB signaling (21, 22). Although TNF inhibitors can be effective for this group of diseases, NF-κB can also be activated by TNF-independent agonists including IL-1β. Accordingly, some patients with NF-κB associated autoinflammatory diseases require treatment with other immunomodulators, including IL-1 pathway inhibitors (3, 18, 23).

**Figure 3:**
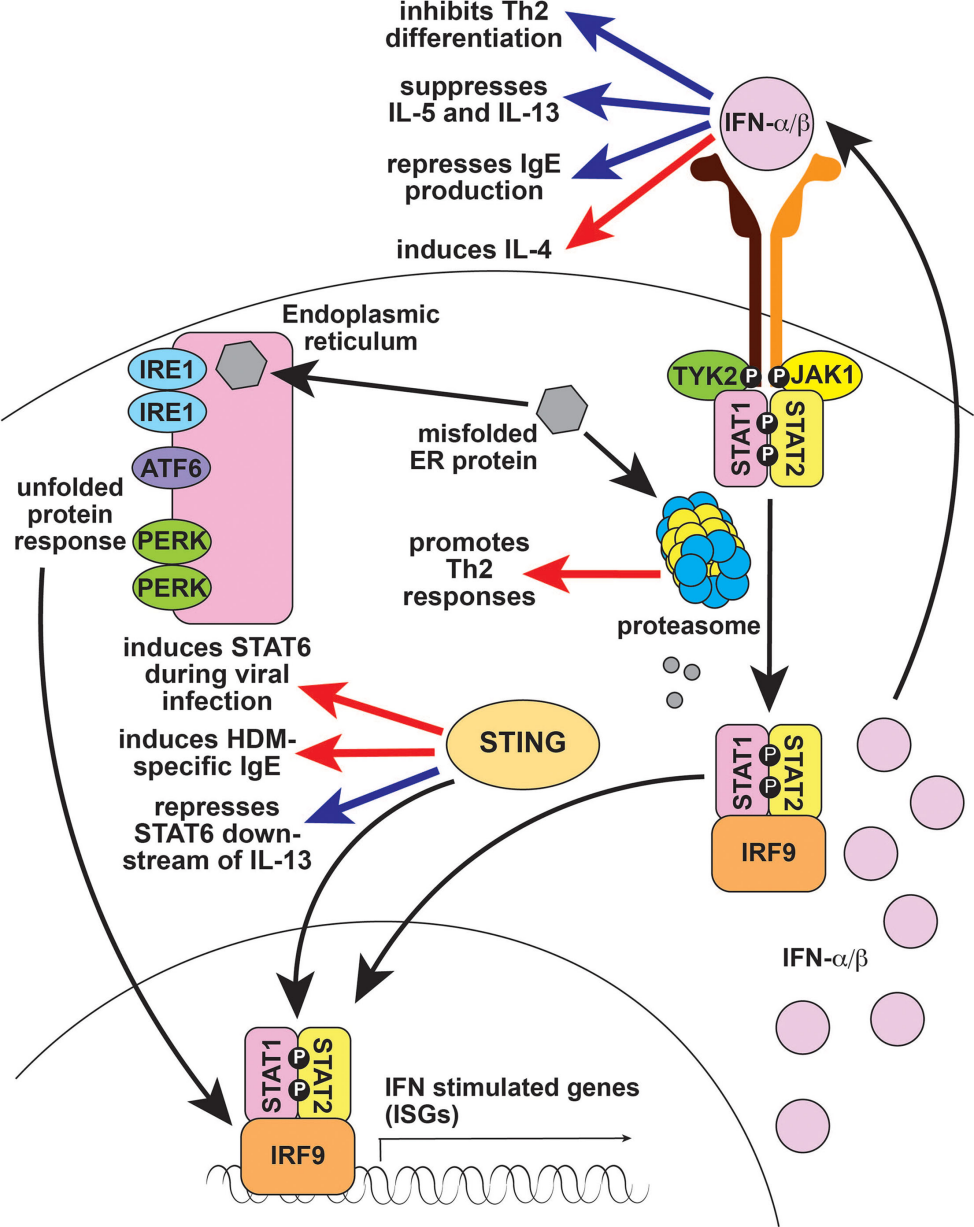
Type 1 interferon signaling in type 2 immune responses. Type 1 IFNs like IFN-α and IFN-β largely suppress type 2 responses (blue arrows), although they are reported to induce IL-4 (red arrow), which can enhance type 2 immunity. The proteasome is important for processing and degrading misfolded endoplasmic reticulum proteins; defects cause unfolded proteins to accumulate, resulting in Type 1 IFN production. The proteasome also regulates antigen processing and presentation which is critical for T cell immunity, including Th2 responses (red arrow). STING is a DNA sensor that activates Type 1 IFN. STING activates STAT6 in response to viral infection and promotes IgE production in response to HDM (red arrow), but also represses IL-13-induced STAT6 activation in subjects with rhinosinusitis (blue arrow). IFN, interferon; ER, endoplasmic reticulum; IRE1, Inositol Requiring Enzyme 1; ATF6, Activating Transcription Factor 6; PERK, PKR-like Endoplasmic Reticulum Kinase; STING, Stimulator of Interferon Genes; TYK2, Tyrosine Kinase 2; JAK1, Janus Kinase 1; STAT1, Signal Transducer and Activator of Transcription 1; IRF9, interferon regulatory factor 9; Th2, T helper 2; IL-4, interleukin 4; IL-5, interleukin 5; IL-13, interleukin 13; IgE, immunoglobulin E, NK, natural killer; HDM, house dust mite.

The Type I interferon (IFN) pathway is important for antiviral immunity and for innate immune functions such as natural killer cell activation and antigen presentation (**Figure 3**) (24). Inborn errors of immunity that cause activation of Type I IFN signaling are termed interferonopathies. Proteasome-associated autoinflammatory syndromes (PRAAS) result from mutations in genes encoding proteasome subunits. Proteasome dysfunction induces the unfolded protein response (UPR), resulting in Type I IFN activation and autoinflammation (25). Several monogenic interferonopathies are caused by mutations in genes that modulate intracellular responses to nucleic acids. For example, mutations in the DNA sensor gene *TMEM173* lead to STING-associated vasculopathy with onset in infancy (SAVI) (26). Mutations in interferon-response genes like *STAT2* can also cause autoinflammation due to overactive signaling downstream of Type I IFN (27, 28).

In addition to these canonical dysregulated pathways, autoinflammation can also be caused by mutations in genes important for other innate immune functions. Deficiency of ADA2 (DADA2) is caused by mutations in *CERC1*, which regulates monocyte differentiation (29). Mutations in complement pathway genes like *CFH, C3*, and *CD46* can cause atypical hemolytic uremic syndrome (3, 30–32). Genes that regulate actin polymerization like *WDR1* and *CDC42* are also important for inflammasome assembly; mutations can therefore cause IL-1β and IL-18-dependent autoinflammation (3, 33, 34). The ripoptosome is a multimeric complex containing RIPK1, FADD, and caspase-8 that is important for regulating the balance between necroptotic and apoptotic cell death; inactivating mutations can therefore cause autoinflammation secondary to increased necroptosis (35–37). Somatic mutations in the ubiquitin-editing gene *UBA1* lead to VEXAS, a treatment-refractory complex autoinflammatory syndrome characterized by activation of multiple immune pathways (38). Finally, a number of complex autoinflammatory diseases including systemic juvenile idiopathic arthritis (sJIA), Behcet’s disease, and periodic fever, aphthous stomatitis, pharyngitis, and cervical adenitis (PFAPA) syndrome are linked to a combination of genetic polymorphisms and environmental factors (39, 40). As increased access to next-generation sequencing accelerates gene discovery, the spectrum of autoinflammatory diseases will likely broaden to comprise new mechanisms of innate immune dysregulation.

### Type 2 Immunity Is Characterized by Allergy-Associated Effector Cytokines and Cells

**Figure 4:**
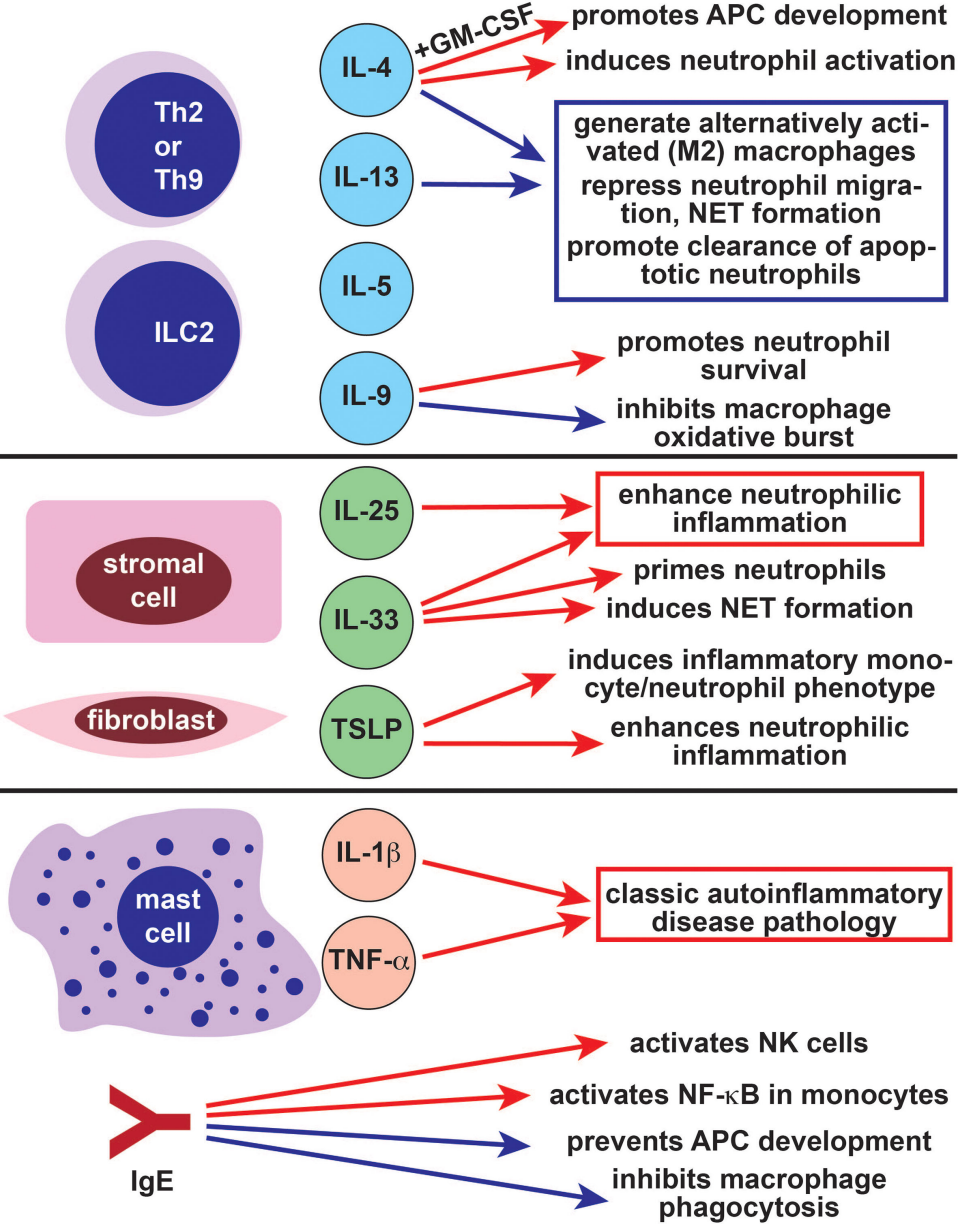
The role of type 2 immune cells and cytokines in autoinflammatory cells and pathways. The Th2- and ILC2-derived cytokines IL-4 and IL-13 largely suppress autoinflammatory pathology by inducing the differentiation of anti-inflammatory M2 alternatively activated macrophages and repressing neutrophil migration (blue arrows). IL-4 also has some positive effects on autoinflammatory cells (red arrows), particularly in combination with GM-CSF. Like IL-4, the Th2/Th9/ILC2-derived cytokine IL-9 has both positive (red arrow) and negative (blue arrow) effects on autoinflammatory cells. The alarmins IL-25, IL-33, and TSLP promote autoinflammatory pathology through their effects on neutrophils and monocytes (red arrows). Mast cells also promote classic autoinflammatory pathology by producing IL-1β and TNF-α. The allergy-associated immunoglobulin IgE can both promote (red arrows) and repress (blue arrows) autoinflammatory disease. Th2, T helper 2; ILC2, type 2 innate lymphoid cell, IL-, interleukin-; GM-CSF, granulocyte macrophage colony stimulating factor; NET, neutrophil extracellular trap; APC, antigen presenting cell; TSLP, thymic stromal lymphopoietin; TNF-α, tumor necrosis factor alpha; IgE, immunoglobulin E; NK, natural killer.

Type 2 immunity was originally described as a counter-regulator of Th1-driven immune responses but was subsequently recognized as a distinct immune response with important roles in antihelminth defense, allergy, and wound repair (4, 5). Type 2 immunity is most commonly associated with Th2 cells and their hallmark effector cytokines IL-4, IL-5, and IL-13. However, type 2 inflammation is mediated by many other cell types including alternatively activated macrophages, type 2 innate lymphoid cells (ILC2), eosinophils, basophils, mast cells, and immunoglobulin E (IgE) secreting plasma cells (8). In addition to Th2-effector cytokines, type 2 immune cells secrete and respond to IL-9, IL-33, IL-25, and thymic stromal lymphopoietin (TSLP) (**Figure 4**) (4).

Immune responses have evolved to protect against discrete pathogens; in this context, type 2 immunity is critical to host defense against helminth infections. Accordingly, type 2 immune cells are found at barrier surfaces where they promote goblet cell hyperplasia, mucus secretion, and muscle contraction – all of which induce intestinal worm expulsion (4, 8). Many of these protective mechanisms can also promote tissue remodeling, making them important for wound repair after injury (4, 8). Type 2 dependent repair can ultimately result in tissue fibrosis, particularly when these pathways are chronically activated (4). Fibrosis is a highly pathological inflammatory endpoint that can result in significant morbidity and mortality secondary to organ failure. Thus, protective type 2 responses can easily become pathogenic when dysregulated or overactivated.

Consistent with the reciprocal inhibition seen for Th1 and Th2 cells, type 2 immune responses can also protect from autoimmune inflammation. This has largely been described in the context of murine inflammatory models, where Th2 cells and type 2 cytokines ameliorate autoimmune arthritis and encephalitis (8, 41, 42). However, the role of type 2 immunity in human autoimmune disease is complex: the type 2 effector cytokines IL-13 and IL-9, for example, are both thought to promote inflammation in patients with ulcerative colitis and psoriasis (43–46). Th2 cells and IgE can both promote kidney inflammation in patients with systemic lupus erythematosus (47, 48).

Allergic disorders make up the largest group of human diseases characterized by type 2 dysregulation and include asthma, atopic dermatitis, food allergy, and allergic rhinitis (2). Immunologically, allergy is caused by an exaggerated type 2 response to foreign antigens. However, many allergy-associated clinical syndromes have forms in which allergic sensitization cannot be demonstrated (49, 50). In some cases, this might be due to primary dysregulation of type 2 inflammatory cells and mediators. For example, some patients with late-onset eosinophilic asthma are thought to have primary dysregulation of ILC2, which produce type 2 cytokines independent of antigenic stimulation (51). Patients with NARES (nonallergic rhinitis with eosinophilia syndrome) are thought to have a primary eosinophilic disorder in at least some cases (50). In other cases, non-type 2 mediators can promote symptoms that are clinically indistinguishable from allergen-specific type 2 responses. For example, hormonal rhinitis can mimic allergic rhinitis but is caused by hormone-induced nasal vascular engorgement (50).

## Part 2: The Role of Autoinflammation in Type 2 Immune Responses

### Autoinflammation, Type 2 Immunity, and Clinical Allergy: A Complex Relationship

Type 1 cytokines have long been thought to primarily repress type 2 immunity based on the Th1-Th2 paradigm. Indeed, the type 1 cytokines IFN-γ and IL-12 inhibit Th2 differentiation and type 2 responses to helminth infection (8, 52, 53). However, other autoinflammatory and autoimmune cytokines can amplify type 2 inflammation, worsening type 2-driven pathology (54–56) Additionally, autoinflammatory and autoimmune cytokines can directly promote tissue inflammation, resulting in clinical phenotypes identical to type 2-driven allergic disease (57–59). The heterogeneity of inflammatory mechanisms driving common clinical phenotypes can present substantial barriers to understanding the crosstalk between type 2 inflammation and autoinflammation in human disease. To help address this complexity, one can approach the role of autoinflammation in type 2-mediated disease using the innate immunologic pathways that are used to categorize monogenic autoinflammatory diseases: inflammasomes, TNF-α, Type I IFN, and newer pathways including necroptosis.

### Inflammasomes and Associated Cytokines in Type 2 Immunity

The pyrin inflammasome does not appear to have a major role in type 2 immune responses, and a recombinant pyrin domain was found to attenuate allergic inflammation in mice by suppressing NF-κB activation (60). By contrast, NLRP3 directly promotes Th2 differentiation independent of its inflammasome function by transcriptionally inducing *Il4* in conjunction with IRF4 (**Figure 1**) (61). The NLRP3 inflammasome can also trigger a Th2-biased response in the context of both infection and allergic inflammation (**Figure 1**) (62–64). NLRP3 activation in bronchial epithelial cells promotes allergic lung inflammation, whereas activation in keratinocytes promotes eczema (63, 65). By contrast, Helicobacter pylori gastric infection protects from allergic asthma by activating NLRP3 in proximal dendritic cells (66). Similarly, helminths induce NLRP3, which then acts as a brake on type 2 responses *via* both inflammasome-independent and inflammasome-dependent mechanisms (**Figure 1**) (67–69). Taken together, these studies suggest that NLRP3 activation may primarily suppress type 2 responses to pathogens but promote dysregulated type 2 responses to environmental allergens.

The end products of inflammasome activation, IL-1α, IL-1β and IL-18, can also regulate type 2 immunity. Single nucleotide polymorphisms (SNPs) in *IL1A, IL1B*, and *IL1R1* are all linked to asthma; accordingly, IL-1α and IL-1β both exacerbate murine allergic airway inflammation (**Figure 1**) (70–75). Type 2 immune cells like eosinophils and mast cells can release IL-1β, airway epithelial cells stimulated with the house dust mite (HDM) allergen can release IL-1α, and IL-1β can be found in allergic tissues, further suggesting that IL-1 has a role in type 2 responses (76–79). This hypothesis is supported by the observation that IL -1β enhances inflammatory Th2 differentiation and helps induce the differentiation of Th9 cells (**Figure 1**) (55, 80–82). IL-1β is also capable of regulating various type 2 innate effector cells to promote tissue inflammation. For example, IL-1β activates human ILC2s in the presence of IL-2, inducing proliferation and effector cytokine production (83, 84). IL-1β also induces histamine release from basophils and mast cells, and histamine enhances IL-1β release, which can induce a positive feedback loop (**Figure 1**) (85, 86). Eosinophils and mast cells stimulated with IL-1β produce IL-9, further supporting the hypothesis that IL-1β can enhance type 2 immune responses to promote allergic pathology (**Figure 1**) (87, 88). A pathogenic role for IL-1 signaling in allergy is further supported by a number of clinical studies demonstrating the efficacy of IL-1 pathway inhibitors in asthma and atopic dermatitis (89–91). Several larger randomized controlled clinical trials have been planned to follow up these encouraging observations but were halted early due to patient recruitment – particularly in light of the ongoing COVID-19 pandemic (NCT01122914, NCT04035109, NCT03513458).

Like IL-1β, IL-18 is reported to enhance Th2 differentiation and T-cell-derived IL-13 production (**Figure 1**) (54, 69). This effect is IL-4-dependent and may be because IL-18-induces IL-4 production or because it increases T cell sensitivity to IL-4 (54). IL-18 also induces IL-13 in natural killer (NK) cells and in basophils, suggesting that it may contribute to the innate arm of type 2 immune responses (**Figure 1**) (92, 93). In addition to IL-13, IL-18 also induces histamine from basophils and can promote eosinophil development and maturation in combination with IL-5 (94, 95). However, IL-18 can also repress type 2 responses *in vivo*. IL-18-deficient mice develop enhanced allergen-induced eosinophilia, and IL-18-deficient mice are protected from helminth infections (**Figure 1**) (96, 97). This suggests that the role of IL-18 in type 2 immunity may be context-dependent. Indeed, IL-18 can promote either Th1 or Th2 differentiation depending on genetic background and cytokine milieu (98). Similarly, IL-18 represses allergic pathology and IgE production in combination with IL-12 but induces both of these in the absence of IL-12 (95, 99, 100).

### TNF-α and NF-κB Signaling in Type 2 Immunity

The inflammatory cytokine TNF-α has a role in both innate and adaptive immunity, underlying the efficacy of TNF inhibitors in patients with autoimmune conditions like rheumatoid arthritis (RA) and autoinflammatory conditions like Deficiency of ADA2 (DADA2) (101, 102). TNF-α and other TNF superfamily cytokines promote the differentiation of Th9 cells, suggesting that they may enhance type 2 immune responses (**Figure 2**) (103, 104). Many TNF superfamily cytokines are costimulatory molecules that more generally modulate division, survival, and activation in T cells. Several of these positively regulate of Th2 differentiation and function due to their role in costimulation (105). TNF-α also enhances the effect of IL-4 on eosinophils and enhances Th2- mediated responses at mucosal sites (**Figure 2**) (56, 106). This may be in part due to effects on non-immune cells that promote type 2 responses. For example, TNF-α and IL-1β synergize to promote airway hyperresponsiveness, which might partly underlie the role of TNF-α in asthma (**Figure 2**) (107, 108). The TNF-α inhibitor etanercept initially showed promise for severe refractory asthma, but a subsequent trial failed to show efficacy (107, 109). Clinical development was ultimately halted due to an increased rate of serious adverse effects, most notably respiratory infections, in a phase 2 trial of golimumab (110). Etanercept and infliximab are reported efficacious for the treatment of atopic dermatitis and have been used as an off-label treatment for severe disease (111, 112).

NF-κB signaling has long been known to play a role in type 2 immune responses, Th2 differentiation, IgE production, and the function of innate type 2 effectors like eosinophils, ILC2s, and mast cells (10, 113–115). Inactivating mutations in NF-κB pathway genes like *CARD11* and *CARD14* cause monogenic immune dysregulatory syndromes that include allergic phenotypes, indicating that physiologic NF-κB signaling can suppress type 2 pathology (**Figure 2**) (116, 117). The clinical phenotype of *CARD14* loss-of-function (LOF) is particularly interesting in the context of autoinflammation, because activating *CARD14* mutations cause a monogenic autoinflammatory disease (22). Similarly, *NOD2 (CARD15)* LOF polymorphisms are associated with an increased risk of clinical allergy and inflammatory bowel disease (IBD), whereas activating mutations cause the autoinflammatory disease Blau syndrome (**Figure 2**) (21, 118, 119). However, NOD2 also induces the type 2 cytokine TSLP, promotes ILC2 expansion, and induces eosinophil activation (**Figure 2**). These studies suggest that, in some cases, NF-kB signaling is primarily an inducer of type 2 immune responses.

The autoinflammation-associated NF-κB signaling repressor A20 (*TNFAIP3*) inhibits airway epithelial cytokine production in response to endotoxin, suppressing type 2 responses to HDM and preventing allergic asthma (**Figure 2**) (120). A20 also has a cell-intrinsic anti-inflammatory role in mast cells, inhibits intestinal Th2 responses, and prevents Th17 differentiation in response to HDM (**Figure 2**) (121–123). These observations may explain the negative associations of *TNFAIP3* expression with allergic asthma, chronic rhinosinusitis, atopic dermatitis, and food allergy (124–127). The SHARPIN protein (Shank-interacting protein like 1) is a part of the LUBAC (linear ubiquitin chain assembly complex), which promotes NF-κB activation and is linked to autoinflammation and complex immune dysregulation. SHARPIN promotes regulatory T cell function, so deficiency promotes systemic inflammation (128). Additionally, SHARPIN deletion causes lymphocyte-independent eosinophilic esophagitis, and keratinocyte-specific deletion causes eosinophilic dermatitis (**Figure 2**) (129–131).

### Type I IFN Signaling in Type 2 Immunity

Broadly, type I IFNs inhibit type 2 immune responses: they suppress IL-5 and IL-13 production, inhibit GATA3-dependent Th2 differentiation, and block B cell isotype switching to IgE (**Figure 3**) (24, 132–135). Accordingly, recombinant IFN-α is used to treat Idiopathic Hypereosinophilic Syndromes and Eosinophilic Granulomatosis with Polyangiitis (136, 137). Type I IFNs are also thought to play a role in asthma, where deficiency leads to increased viral infection and enhanced Th2 differentiation, worsening disease (138). However, type I IFNs are also reported to induce IL-4 production and to promote murine eosinophilic rhinosinusitis, possibly by increasing eosinophil recruitment (**Figure 3**) (134, 139). It remains to be determined whether these functions have any role in promoting type 2 immunity-related human diseases.

In addition to modulating type I IFNs through the unfolded protein response, the proteasome is important for antigen processing and presentation (140). Consequently, defects in the proteasome result in a general loss of T-cell-dependent immunity. Taken together with the antagonistic role of type I IFN on type 2 responses, it is not surprising that defects in the proteasome are associated with reduced Th2 responses (**Figure 3**) (141). By contrast, the DNA sensor STING activates the type 2 associated signaling molecule STAT6 in response to viral infection, although the result is enhanced antiviral immunity rather than a type 2 immune response (**Figure 3**) (142). STING also promotes HDM-induced IgE production by enhancing the function of T follicular helper cells (**Figure 3**) (143). However, STING represses IL-13-induced STAT6 phosphorylation in subjects with rhinosinusitis by increasing expression of the STAT6 inhibitor SOCS1 (suppressor of cytokine signaling 1) (**Figure 3**) (144). SOS1 induction may also underlie the observation that STING signaling in ILC2s promotes a phenotypic shift to Type 1 ILC (ILC1) during lung inflammation (145). Taken together, these studies suggest that the role of STING in type 2 immunity is complex and context dependent.

### Other Autoinflammation-Associated Pathways in Type 2 Immunity

Cytoskeletal regulators that play a role in inflammasome activation, like CDC42 and WDR1, also play a role in adaptive immunity and nonhematopoietic cells. CDC42 is activated by the atypical guanine nucleotide exchange factor DOCK8, which is linked to autosomal recessive hyper-IgE syndrome (33). CDC42 signaling is also important for mast cell and eosinophil function, and CDC42-deficient invariant natural killer T cells have a defect in IL-4 secretion because CDC42 degradation induces IL-4 secretion in response to lipid antigens (146). Complement activation promotes Th1 differentiation and function, which can indirectly repress type 2 responses, but is not thought to directly regulate Th2-driven responses (147). The complement system can activate innate type 2 effectors like eosinophils and mast cells, however, and may therefore promote some type 2 associated pathology (148, 149). Environmental allergens can activate the ripoptosome to trigger type 2 inflammation through RIPK1 and caspase 8, which shunt cells away from necroptosis and towards apoptosis (**Figure 2**) (36, 150, 151). Caspase-8 can also promote allergic pathology by directly activating IL-1 cytokines (152). However, caspase 8 prevents type 2 immune responses to *Trypanosoma cruzi* infection, leading to increased parasitemia and chronic infection (150). Caspase-8 also promotes epithelial keratinocyte cohesion, so that epidermal-specific deficiency causes a spontaneous eczematoid dermatitis (153). Thus, the effect of the ripoptosome on type 2 immune responses may be context-dependent as for other autoinflammatory mediators.

## Part 3: The Role of Type 2 Immunity in Autoinflammatory Cells and Pathways

### Th2 and ILC2-Derived Cytokines in Autoinflammatory Cells and Pathways

The type 2 cytokines IL-4 and IL-13 have long been studied as modulators of innate immune function due to their role in the generation of alternatively activated macrophages (M2) (**Figure 4**) (154). In contrast to classical activation, which is induced by IFN-γ and characterized by type 1 cytokine production and microbial killing, alternative activation causes macrophages to develop an immunoregulatory function. M2 macrophages are not efficient killers of invading pathogens but produce growth factors and extracellular matrix components, making them important for wound healing (154). They also can generate or maintain type 2 immune responses. In the context of alternative activation, IL-4 promotes tissue resident macrophage activation and accumulation (155). Exposure to IL-4 in combination with GM-CSF (granulocyte-monocyte colony stimulating factor) causes peripheral monocytes to function as antigen presenting cells (**Figure 4**) (156). These monocyte-derived cells phenotypically resemble inflammatory dendritic cells rather than inflammatory macrophages (157). Indeed, the inflammatory macrophage phenotype is promoted by classical activation and inhibited by alternative activation (158). This may be because IL-4 inhibits NF-κB and inflammasome activation in macrophages, reducing responsiveness to lipopolysaccharide (158).

IL-4 also has a role in neutrophil biology and can even be produced by neutrophils (159). While IL-4 can induce neutrophil activation and phagocytosis, it also inhibits neutrophil migration (**Figure 4**) (160, 161). IL-4 also represses the formation of neutrophil extracellular traps (NETs), an important mechanism used for pathogen killing (162). Like IL-4, IL-13 inhibits neutrophil migration to inflamed tissues, although IL-13 also enhances production of several neutrophil effector proteins including IL-8 (163, 164). Finally, the IL-4 and IL-13 activated signaling molecule STAT6 is importance for clearance of apoptotic neutrophils, which promotes resolution of inflammatory responses (165). Taken together, these data suggest that type 2 cytokines primarily repress pathways associated with autoinflammation in macrophages and neutrophils.

Like IL-4 and IL-13, the type 2 cytokines IL-5 and IL-9 are derived primarily from T helper cells and ILC2s. While neither IL-5 nor IL-9 is implicated in alternative activation of macrophages, both cytokines can modulate the function of monocytes and neutrophils. IL-5 receptor is expressed on neutrophils, including airway-resident neutrophils from asthma patients, although its function in neutrophils is not well characterized (166, 167). IL-5 indirectly regulates dendritic cells by inducing eosinophils, which repress plasmacytoid dendritic cell derived type I IFN production (168). IL-9 represses autoinflammation-associated responses by inhibiting oxidative burst and TNFα release in LPS-stimulated human monocytes and alveolar macrophages (**Figure 4**) (169, 170). However, IL-9 can also promote neutrophil survival and neutrophil-derived IL-8 release, enhancing type 1 inflammatory responses (171, 172). This suggests that the role of IL-9 in autoinflammation is complex and context-dependent.

### Alarmins in Autoinflammatory Cells and Pathways

Type 2 innate cytokines, or alarmins, are produced by epithelial cells, endothelial cells, stromal cells, and fibroblasts in response to injury. These alarmins include IL-25, IL-33 and TSLP; they activate ILC2, Th2, eosinophils, mast cells, and other type 2 effectors (5, 10, 173, 174). Because activated ILC2 and Th2 cells produce large amounts of IL-4 and IL-13, alarmins can indirectly promote ILC2-dependent immunosuppressive functions in neutrophils (175, 176). In some cases, alarmins can also directly regulate neutrophils and monocytes. For example, IL-33 primes neutrophils so that they are rapidly recruited to sites of infection and inflammation, whereas IL-25 promotes neutrophilic airway infiltration (**Figure 4**) (177–180). IL-33 overexpression causes spontaneous neutrophilic arthritis and sterile inflammation possibly due to increased NET formation (181). The alarmin TSLP enhances neutrophilic inflammation and induces a proinflammatory phenotype in circulating monocytes and neutrophils (**Figure 4**) (182, 183). Further supporting its role in neutrophil-mediated host defense, TSLP enhances neutrophilic microbicidal activity against methicillin-resistant *Staphylococcus Aureus* (184). Together, these data suggest that type 2 alarmins can promote autoinflammatory pathology in some contexts.

### Mast Cells and High Affinity IgE Receptor in Autoinflammatory Cells and Pathways

Mast cells produce IL-1β; which is cleaved and activated by caspase 1, caspase 8, and serine proteases (**Figure 4**) (185). Mast cell IL-1β production is NLRP3-dependent, suggesting that mast cells may have a role in NLRP3-associated autoinflammatory processes. Accordingly, patients with *NLRP3* mutations develop cold-induced histamine-independent urticariform lesions, and mast cells are a major source of IL-1β in affected skin (77, 79). Mast cells also produce IL-1β in patients with the adult-onset autoinflammatory disease Schnitzler’s syndrome, in subjects with chronic recurrent multifocal osteomyelitis, and have been found in inflamed joints from patients with FMF (186–188). In mice, mast cells promote sterile joint and central nervous system inflammation (185, 189). Mast cell derived TNF-α induces urticariform rashes in patients with *NLRP3* mutations, although the role of mast cell derived TNF-α in other autoinflammatory diseases is not known (**Figure 4**) (190).

IgE is a critical inducer of many type 2 effector cells, including mast cells, through its high affinity receptor Fc epsilon RI. Fc epsilon RI is also expressed and functional in several type 1 innate effector cells. IgE crosslinking suppresses monocyte function by blocking phagocytosis and preventing differentiation into dendritic cells (**Figure 4**) (191, 192). Simultaneously, engagement of Fc epsilon RI activates NF-κB in monocytes and dendritic cells, which promotes secretion of IL-6, IL-10, and TNF-α (192, 193). Macrophage Fc epsilon RI engagement also reprograms alternatively activated tumor-resident macrophages to be more proinflammatory, enhancing their antitumoral functions (194). The functions of IgE and its receptor are not as well characterized in other type 1 innate cells. However, Fc epsilon RI is expressed in both dendritic cells and neutrophils, where it delays neutrophil apoptosis (195–197). IgE can also activate NK cells through the lower affinity Fc gamma RIII receptor (198). Future studies will be needed to further characterize the roles of IgE and its receptors in autoinflammation-associated innate immune cells.

## Part 4: The Epidemiology of Allergy in Autoinflammatory Diseases

### The Epidemiology of Allergy in Monogenic Autoinflammatory Diseases

One way to investigate the interaction between autoinflammatory pathways and type 2 immunity is to investigate the prevalence of allergic clinical and immunological phenotypes in subjects with monogenic autoinflammatory diseases (**Table 1**). Because single gene mutations promote activation of discrete innate pathways, this approach can assess the *in vivo* roles of dysregulated autoinflammatory pathways in regulating human type 2 immune responses (199). This question has been most extensively studied in FMF, perhaps because it was the first autoinflammatory disease to be linked to a causative gene (16) (**Table 1**). Several studies have suggested that FMF protected against asthma and atopy, potentially due to protective linkage of *MEFV* with asthma associated genes like *IL4RA* (199–201). Although one study suggested that Turkish FMF patients may have elevated total serum IgE relative to healthy volunteers, this result was not seen in other cohorts, where there was a trend towards reduced serum IgE (199, 201, 202). Taken together, these results suggest that activation of the pyrin inflammasome attenuates human type 2 immune responses.

**Table 1:** Associations of monogenic autoinflammatory diseases with type 2 clinical and immunological phenotypes.

Disease	Gene(s)	Type 2 Phenotype
FMF	*MEFV*	Reduced prevalence of asthma (199–201) Increased prevalence of rhinosinusitis (199) Elevated total serum IgE relative to healthy volunteers (202) Reduced total serum IgE relative to healthy volunteers (201) Reduced mean absolute eosinophil count relative to healthy volunteers (199)
CAPS	*NLRP3*	Increased prevalence of hypereosinophilia, asthma, eczema, and rhinosinusitis relative to healthy volunteers (199, 203) Increased mean absolute eosinophil count relative to healthy volunteers (199) Th2 cell expansion (199)
NOCARH	CDC42	Mild hypereosinophilia and hyper-IgE (204)
TRAPS	*TNFRSF1A*	Increased prevalence of allergic rhinitis, eosinophilic GI disease relative to healthy volunteers (199) Th2 cell expansion (199)
CANDLE	*POMP*	Increased prevalence of eczema, eosinophilic GI disease relative to healthy volunteers (199)
	*PSMA3*	Reduced prevalence of asthma relative to healthy volunteers (199)
	*PSMB10*	Reduced mean absolute eosinophil count relative to healthy volunteers (199)
	*PSMB4*	
	*PSMB8*	
	*PSMB9*	
	*PSMG2*	
DADA2	*CERC1*	Increased prevalence of eczema, allergic rhinitis relative to healthy volunteers (199)
		Reduced mean absolute eosinophil count, total serum IgE relative to healthy volunteers (199)
HA20	*TNFAIP3*	Increased prevalence of eczema, allergic rhinitis, eosinophilic GI disease relative to healthy volunteers (199)
		Th9 cell expansion (19, 199)
HIDS	*MVK*	Increased prevalence of allergic rhinitis, eosinophilic GI disease relative to healthy volunteers (199)
		Reduced total serum IgE, mean absolute eosinophil count relative to healthy volunteers (199)
PAPA	*PSTPIP1*	Reduced mean absolute eosinophil count relative to healthy volunteers (199)

By contrast, the autoinflammatory disease CAPS, caused by *NLRP3* mutations, is associated with peripheral eosinophilia and eosinophilic skin infiltration (199, 203) (**Table 1**). Eosinophilia correlates with CAPS disease activity, suggesting that *NLRP3* activation promotes eosinophilia (199). This is consistent with the role of NLRP3 and IL-1β in promoting the differentiation and function of type 2 effectors like Th2 cells, mast cells, and eosinophils. CAPS is also characterized by an increased prevalence of eczema, asthma, and allergic rhinitis relative to both the general population and FMF (199). This is consistent with the observation that NLRP3 activation exacerbates murine models of asthma and eczematous dermatitis (63, 65). Finally, the urticariform lesions of CAPS are characterized by IL-1β and TNF-α producing mast cell infiltration, once again linking the NLRP3 inflammasome to type 2 effector activation in humans (77, 79, 190). Overall, these results suggest that in humans, constitutive activation of the NLRP3 inflammasome promotes type 2 immune responses. Because helminth infections are extremely uncommon in countries with highly developed CAPS cohorts, it remains to be determined whether NLRP3 activation suppresses type 2 responses to pathogens in humans, as it does in murine models (67–69).

CDC42 is a plasma membrane associated GTPase involved in diverse processes including cell division, phagocytosis, and epithelial cell morphology (204). Mutations are linked to NOCARH (neonatal onset of pancytopenia, autoinflammation, rash, and episodes of HLH) an IL-1-responsive autoinflammatory disease with features of macrophage activation syndrome (MAS) (33, 205). CDC42 alternates between an inactive cytosolic form and an active plasma membrane bound form; mutations affecting trafficking alter the subcellular localization independent of the protein’s activation state (33). This, in turn, alters the partners that bind to CDC42, ultimately leading to NF-κB overactivation and autoinflammation (204). In addition to autoinflammation, one patient with NOCARH also developed mild hypereosinophilia and hyper-IgE, although no clinical allergic diagnoses were reported (204) (**Table 1**). As additional patients are identified, careful phenotyping will be needed to determine whether type 2 immune activation is a common feature of NOCARH.

Far less is known about the prevalence of type 2 immune activation in other autoinflammatory diseases. In one systematic population study, clinical diagnoses of allergic rhinitis were highly prevalent in almost all autoinflammatory diseases, including FMF (199). This included diseases with reduced clinical laboratory markers of type 2 inflammation relative to the general population, like HIDS and DADA2. This might be because autoinflammation-associated cytokines like IL-1β and TNF-α can promote sinus mucosal thickening independent of type 2 immune activation (206). Thus, in some cases, autoinflammatory pathology may mimic type 2 associated disease, and this may be a potential confounder in epidemiologic studies.

### The Epidemiology of Allergy in Complex Autoinflammatory Diseases

Unlike their monogenic counterparts, complex autoinflammatory diseases are linked to multiple genetic and environmental factors that contribute to their pathogenesis. Behcet’s disease is a heterogeneous and complex autoinflammatory disease that manifests with orogenital ulcers, pustular skin disease, arthritis, eye disease, gastrointestinal inflammation, and vascular complications (207). Genetic studies have identified a number of risk alleles that overlap with both recurrent aphthous stomatitis and PFAPA syndrome, allowing the three syndromes to be grouped together as Behcet’s spectrum disorders (39). Amongst the susceptibility loci shared by Behcet’s spectrum disorders are multiple genes associated with Th1-driven immunity, such as *STAT4* and *IL12A* (39). Th1 cells are thought to repress Th2 cells, and perhaps for this reason patients with Behcet’s disease were found in several studies to have lower rates of allergic sensitization and lower IgE levels than the general population (201, 208) (**Table 2**). However, a separate group of studies reported increased rates of atopy and elevated levels of type 2 cytokines in subjects with Behcet’s spectrum disorders, particularly children with PFAPA (209–212). Moreover, the IgE-blocking monoclonal antibody has been reported to alleviate symptoms and reduce autoinflammation in one subject with concurrent Behcet’s disease and asthma (213) (**Table 2**). These disparate findings may be partly due to the genetic and phenotypic heterogeneity of patients with Behcet’s spectrum disorders, which can vary substantially between cohorts with different ancestries (207).

**Table 2:** Associations of complex autoinflammatory diseases with type 2 clinical and immunological phenotypes.

Disease	Type 2 Phenotype
Behcet’s spectrum disorders (Behcet’s disease, PFAPA, aphthous stomatitis)	Lower rates of allergic sensitization and lower IgE levels than the general population (201, 208)
	increased rates of atopy and elevated levels of type 2 cytokines PFAPA (209–212).
	Clinically responds to omalizumab (anti-IgE) (213)
sJIA/AOSD	Atopy is a risk factor for severe disease in sJIA (215)
	Positive association of AOSD with elevated serum IgE, IL-4, and clinical atopy (case reports) (216, 217)

Systemic juvenile idiopathic arthritis (sJIA) is another complex autoinflammatory disease with genetic and phenotypic links to both autoinflammation and autoimmune inflammation (40). Children with allergic disease were found to be at a higher risk of developing JIA in a Taiwanese cohort, although sJIA was not differentiated from other forms (214). Atopy may also be a risk factor for increased disease severity in sJIA, although this has only been investigated in one small prospective study (215). Adult-onset Still’s disease (AOSD) is an adult-onset clinical syndrome that phenotypically resembles sJIA (216). Cases of AOSD have been reported in association with elevated serum IgE, IL-4, and clinical atopy, but the prevalence of these features has not yet been systematically investigated (216, 217).

## Conclusions

Although autoinflammation and type 2 immunity have traditionally thought to counter-regulate each other, a growing body of literature demonstrates that the relationship between type 1 and type 2 immune responses is more nuanced than this canonical view would suggest. Some autoinflammatory cytokines, like IL-1β and TNF-α; enhance the differentiation and function of type 2 effector cells and exacerbate allergic pathology. Others, like type 1 IFN, largely repress type 2 inflammation but can promote type 2 cytokine production in certain contexts. And some autoinflammatory signaling molecules like NLRP3 may constrain type 2 responses in the context of parasitic infection, while inducing type 2 immunity in the setting of allergic inflammation. These observations suggest that the role of autoinflammation in type 2 immunity may rely on a broad array of genetic and environmental factors involved in driving the immune response.

Similarly, the role of type 2 immunity in the pathogenesis of autoinflammation is complex and context-dependent. While Th2- and ILC2-derived type 2 cytokines like IL-4 and IL-13 generally repress type 1 inflammation, they can promote neutrophil activation in certain context. Moreover, alarmins like IL-33 and TSLP clearly induce autoinflammatory effectors like neutrophils and monocytes, causing local and systemic inflammation. Mast cells, which are generally considered type 2 effectors, have a clear role in *NLRP3*-associated autoinflammatory diseases and may play a role in diseases linked to other genes like *MEFV*. Future investigation will be required to determine the roles played by type 2 cytokines and effectors in modulating pathology in subjects with monogenic and complex autoinflammatory diseases.

Clinical epidemiology studies in patients with autoinflammatory diseases paint a similarly nuanced picture. Given the role of *NLRP3* in promoting allergic pathology, for example, it is not surprising that the phenotypic spectrum of CAPS comprises eosinophilia and clinical allergy in addition to systemic autoinflammation. In other syndromes, autoinflammation appears to have a negative effect on type 2 immunity – most notably for FMF – although the mechanisms are not well-defined. Finally, in some cases, it appears that autoinflammatory pathology can mimic allergic disease, causing a phenotype that is indistinguishable from clinical allergy but that is not mediated by type 2 effectors. These observations have clinical implications for subjects with autoinflammatory diseases, where type 2 directed therapies have been reported effective in some cases. They may also have repercussions for subjects with clinical allergy-associated diagnoses like asthma, where non-allergic endotypes are unlikely to respond to type 2 directed therapies. The ability of autoinflammatory cytokines and mediators to both potentiate and clinically phenocopy type 2 pathology suggests that some of these patients might benefit from autoinflammation-directed treatments. In the future, dissecting the interactions between these two not-so-separate arms of the immune response should help to refine our understanding of – and improve treatments for – monogenic and complex immune dysregulatory disorders.

## Author Contributions

MS, AS, and DS organized and composed the manuscript. MS created tables. DS created figures and provided supervision. All authors contributed to the article and approved the submitted version.

## Funding

This work was supported by the NIH/NIAID intramural research program (grant no. 1ZIAAI001251).

## Conflict of Interest

The authors declare that the research was conducted in the absence of any commercial or financial relationships that could be construed as a potential conflict of interest.

## Publisher’s Note

All claims expressed in this article are solely those of the authors and do not necessarily represent those of their affiliated organizations, or those of the publisher, the editors and the reviewers. Any product that may be evaluated in this article, or claim that may be made by its manufacturer, is not guaranteed or endorsed by the publisher.
